# New Primers for Discovering Fungal Diversity Using Nuclear Large Ribosomal DNA

**DOI:** 10.1371/journal.pone.0159043

**Published:** 2016-07-08

**Authors:** Asma Asemaninejad, Nimalka Weerasuriya, Gregory B. Gloor, Zoë Lindo, R. Greg Thorn

**Affiliations:** 1 The University of Western Ontario, Department of Biology, London, Ontario, Canada N6A 5B7; 2 The University of Western Ontario, Department of Biochemistry, London, Ontario, Canada N6A 5B7; Georg-August-University of Göttingen Institute of Microbiology & Genetics, GERMANY

## Abstract

Metabarcoding has become an important tool in the discovery of biodiversity, including fungi, which are the second most speciose group of eukaryotes, with diverse and important ecological roles in terrestrial ecosystems. We have designed and tested new PCR primers that target the D1 variable region of nuclear large subunit (LSU) ribosomal DNA; one set that targets the phylum Ascomycota and another that recovers all other fungal phyla. The primers yield amplicons compatible with the Illumina MiSeq platform, which is cost-effective and has a lower error rate than other high throughput sequencing platforms. The new primer set LSU200A-F/LSU476A-R (Ascomycota) yielded 95–98% of reads of target taxa from environmental samples, and primers LSU200-F/LSU481-R (all other fungi) yielded 72–80% of target reads. Both primer sets have fairly low rates of data loss, and together they cover a wide variety of fungal taxa. We compared our results with these primers by amplifying and sequencing a subset of samples using the previously described ITS3_KYO2/ITS4_KYO3 primers, which amplify the internal transcribed spacer 2 (ITS2) of Ascomycota and Basidiomycota. With approximately equivalent read depth, our LSU primers recovered a greater number and phylogenetic diversity of sequences than the ITS2 primers. For instance, ITS3_KYO2/ITS4_KYO3 primers failed to pick up any members of Eurotiales, Mytilinidiales, Pezizales, Saccharomycetales, or Venturiales within Ascomycota, or members of Exobasidiomycetes, Microbotryomycetes, Pucciniomycetes, or Tremellomycetes within Basidiomycota, which were retrieved in good numbers from the same samples by our LSU primers. Among the OTUs recovered using the LSU primers were 127 genera and 28 species that were not obtained using the ITS2 primers, although the ITS2 primers recovered 10 unique genera and 16 species that were not obtained using either of the LSU primers These features identify the new primer sets developed in this study as useful complements to other universal primers for the study of fungal diversity and community composition.

## Introduction

Identification and classification of a wide variety of organisms using DNA sequences has helped overcome many limitations of traditional morphological approaches, including providing faster analyses, resolving convergent morphologies, recognition of closely related or sister species, and the ability to identify cryptic organisms from complex or opaque substrates [1,2]. This has been especially true for the assessment of the fungi for which, as the second most speciose eukaryotic group with complex and often cryptic life histories and convergent morphological characteristics, traditional taxonomic approaches have long been problematic [3,4]. Alongside a growing recognition that fungi have an important role in nutrient mineralization and uptake affecting plant productivity and overall ecosystem process [5,6], there has been growing application of genetic markers for the purpose of identification and community composition analyses [7,8].

However, the utility of DNA as a tool to catalogue biodiversity, resolve phylogenies, or explore patterns in ecological communities depends strongly on choice or design of primers for selecting the appropriate genetic markers [9]. Primer sets need to be general enough to match across all members of a broad taxonomic group, while containing mismatches to non-target taxa, and yet yield a gene product variable enough to distinguish taxa at narrow, preferably species-level, resolution [10,11]. Using DNA for species-level identifications (DNA barcoding sensu [12]) typically relies on short, diagnostic DNA sequences, such as the mitochondrial COI gene for metazoan animals [13], the rbcl+matk combination of protein coding genes for plants and green algae [14], or the ribosomal internal transcribed spacer (ITS) region in fungi [15]. However, ongoing examination of the ITS for fungal diversity has revealed that the ITS region is too variable to align over distantly related taxa, and therefore unable to confidently place sequences at the level of family, order or class for which no closely matching reference sequences exist [16]. Moreover, in some genera of filamentous ascomycetes there is little variation in the ITS region, making this domain undesirable for identification or taxonomic analyses at the species level [17–22]. Overall, fungal identification and taxonomic analyses using ITS remain problematic. The D1 variable region of the large ribosomal subunit [23] is an attractive alternative, since it (often together with D2) has proven useful in species-level identification and phylogenetic reconstruction in various fungal groups [24,25].

An ongoing challenge in diversity studies is the pairing of appropriate genetic markers and targeted primer development that works with contemporary technologies. Next Generation Sequencing (NGS) can capture low abundance DNA and provide detailed analyses of relative abundances in microbial communities [26] at a relatively low cost. Among NGS methods, Illumina MiSeq sequencing is the most effective and extensively used technology globally [16] due to its low rate of error and the lowest cost per million bases [27], but requires short diagnostic regions of 300 base pairs to be effective (http://www.illumina.com/systems/sequencing.html). Unfortunately, no primers have been developed to target the D1 region across a wide diversity of fungi with a short amplicon suitable for the Illumina MiSeq platform. Here we introduce and evaluate two primer sets targeting the D1 region of the large subunit (LSU) of ribosomal DNA to evaluate fungal diversity. We demonstrate how these primers can be applied for discovery of major fungal groups to recover a broad range of fungal taxa with confident higher-level placement (family, order, etc.) and potential species or species-group identification.

## Materials and Methods

### Primer design

Invariate regions representing potential primer sites were first identified in an alignment of 591 sequences of Basidiomycota isolated from soil supplemented with 225 reference sequences from GenBank (National Center for Biotechnology Information [NCBI]) and anchored with the sequence of *Saccharomyces cerevisiae* (Accession #: J01355; [28]). This alignment, from the 5’ end of LSU to position 650 (primer site LR3, [29]) was created in two halves using SINA v1.2.9 [30] and the two files merged using profile:profile alignment in MUSCLE [31]. The alignment was trimmed to the potential primer sites and amplicon region (bases 200–500), and a neighbor-joining tree recovered essentially the same topology and nearly all species-level OTUs obtained using the full alignment (223 of 382 aligned sites were parsimony informative; data not shown).

**Fig 1:**
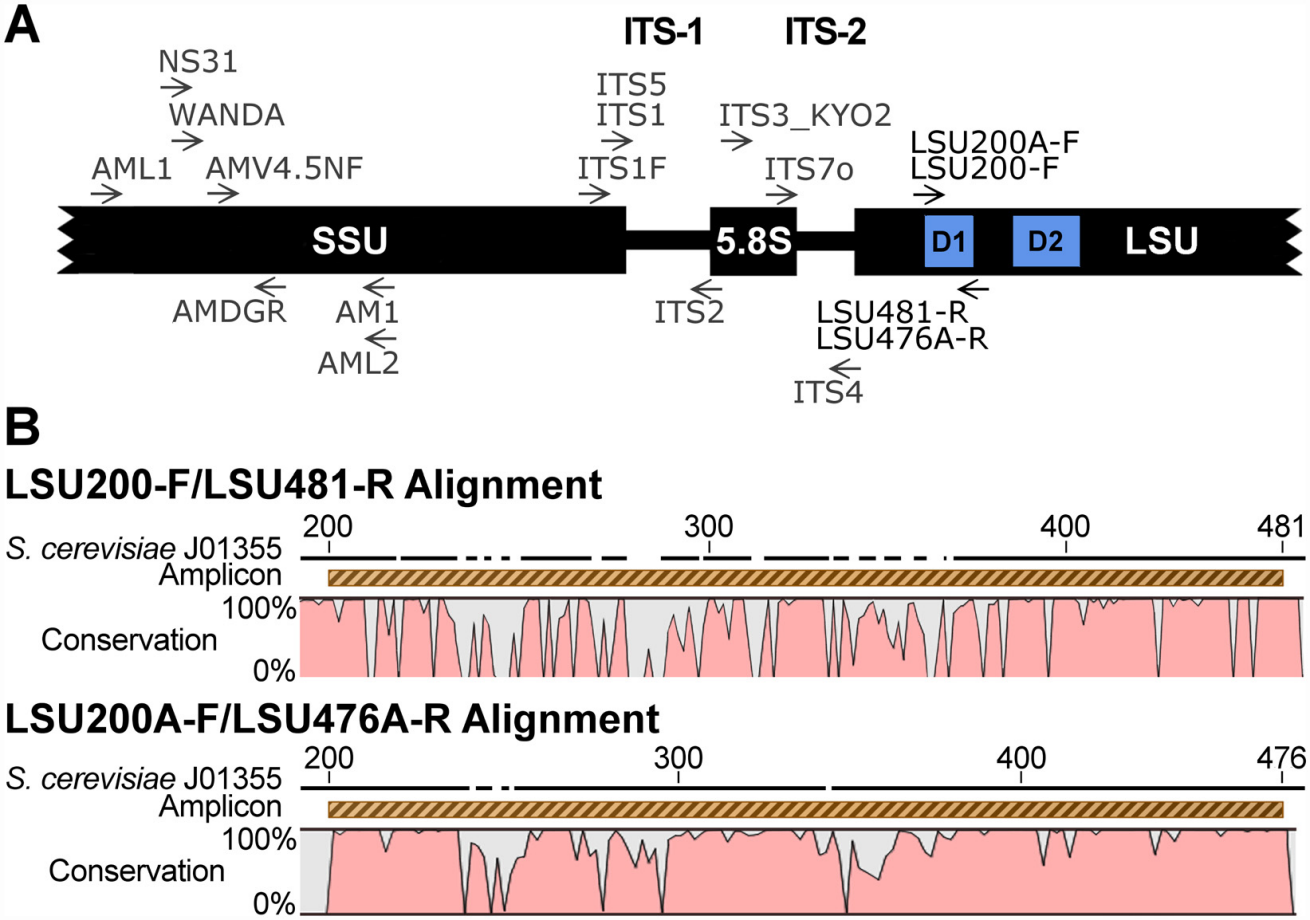
Ribosomal RNA primer map and alignment of LSU200-F/LSU481-R and LSU200A-F/LSU476A-R primers developed in this study. **A**, Approximate location of LSU200-F/LSU481-R and LSU200A-F/LSU476A-R primers in relation to the D1/D2 variable domains within the LSU of *Saccharomyces cerevisiae* J01355 in relation to ITS1, ITS2, ITS4 from White et al. [35], NS31 from Simon et al. [36], AM1 from Helgason et al. [37], AMV4.5N-F and AMDG-R from Sato et al. [38], AML1 and AML2 from Lee et al. [39], WANDA from Dumbrell et al. [40], ITS3_KYO2 from Toju et al. [41], and ITS7o from Kohout et al. [42]. **B**, LSU200-F/LSU481-R and LSU200A-F/LSU476A-R alignments made using a custom reference sequence set, aligned using Muscle v 3.8.31 [31] and visualized using CLC Sequence Viewer (http://www.clcbio.com/), with *S*. *cerevisiae* J01355 included as positional reference.

The region of LSU between bases 150 and 550 from different fungal groups was used to query GenBank using BLAST [32], separately specifying all orders of Agaricomycetes [33] and other Basidiomycota known from soil. The potential 5’ primer at bases 200–220 was recognized as the reverse complement of ITS6-R, previously recommended for amplification of diverse fungi [34], and was modified slightly for greater coverage of fungi and renamed LSU200-F. At the 3’ end, positions 458–481, which did not represent any known primers, were selected for further investigation (Fig 1). These short sequences were used to query GenBank using BLAST, directed to target taxa (Fungi) and non-target taxa (Eukarya, not Fungi).

The primers referred to as LSU200-F and LSU481-R (Table 1) showed nearly complete identity, particularly at their 3’ ends, towards all Basidiomycota, Glomeromycota, Chytridiomycota, Blastocladiomycota, Kickxellomycotina, Mortierellomycotina, Mucoromycotina, and Zoopagomycotina, but not to Ascomycota, and mismatches to other Eukarya (non-target taxa) were numerous. To optimize Ascomycota primers, a separate alignment of Ascomycota was created by querying Entrez for representatives of all major Ascomycota lineages [43] then using BLAST to obtain similar sequences, resulting in an alignment of 131 sequences. Primer LSU200-F had six sites of mismatch with various Ascomycota which were disregarded as they represented either a single base mismatch in the middle of the sequence (e.g., 208A in *Saitoella*, 209C in *Taphrina*) or were mismatches at the 3’ end that occurred only rarely (e.g., 217G in *Neolecta* and 218C in *Taphrina*; but see footnotes to Table 1). For other mismatches, bases 203K, 214C, 215R, and 217Y were altered to match the majority of Ascomycota for the new primer LSU200A-F (Table 1).

**Table 1:** Fungal nuclear large ribosomal primer targets (nu-LSU), with positions numbered relative to *Saccharomyces cerevisiae* (GenBank Accession #: J01355).

Name	Position	Sequence (5’ to 3’)	Nomenclature	Tm	Target Taxa
LSU200-F	200–218	AACKGCGAGTGAAGMGGGA	nu-LSU-200-5′	64	Fungi minus Ascomycota
LSU481-R	462–481	TCTTTCCCTCACGGTACTTG	nu-LSU-481-3′	59	Fungi minus Ascomycota
LSU200A-F^a^	200–218	AACKGCGAGTGAAGCRGYA	nu-LSU-200-5′	63	Ascomycota
LSU476A-R^b^	457–476	CSATCACTSTACTTGTKCGC	nu-LSU-476-3′	59	Ascomycota

^a^ A slight modification (AACKGCGAGTGAAGCRGBM) is recommended to recover all known Ascomycota including Archaeorhizomycetes.

^b^ LSU476A-R has a perfect match with sequences of known Archaeorhizomycetes.

The reverse primer LSU481-R had two pairs of mismatches to most Ascomycota at bases 468–469 and 474–475, which were corrected, and the revised primer LSU476A-R was moved 5 bases 3’-ward for greater specificity against plants and other non-fungal eukaryotes. Both forward and reverse primers were modified on their 5’ ends to include the following: forward and reverse Illumina MiSeq adaptors to allow sequences to bind to the flow cells, a 4 bp random linker (NNNN) to increase the diversity of the first positions sequenced and so enable the instrument to more easily separate clusters and set appropriate fluorescence levels, and unique 8 nt barcode sequences with an edit distance of at least 4 (https://github.com/ggloor/miseq_bin). Primer layout was as follows:

5′–[Illumina Forward Adaptor][NNNN][Barcode][PCR Primer]–3′.

The unique forward and reverse barcode sequences are a paired-end multiplexing approach that allowed us to combine samples within a single Illumina run and bioinformatically separate them back into samples afterwards [44].

We compared our developed primer sets against another primer set currently used in Illumina sequencing (the ITS3_KYO2/ITS4_KYO3 primers, hereafter referred to as “ITS2 primers”) described by Toju et al. [41], where the forward primer is ITS3_KYO2 (5′–GAT GAA GAA CGY AGY RAA–3′), and the reverse primer is ITS4_KYO3 (5′– CTB TTV CCK CTT CAC TCG –3′). These primers target diverse taxonomic groups within Ascomycota and Basidiomycota. Primers were ordered with an Illumina Adaptor, linker, and unique barcode as described above.

### Soil and peat collection

Subarctic soil samples were taken from eight sites within 500 m of the Torngat Mountains Base Camp and Research Station, Kangidluasuk, Labrador, Canada (58.454° N; 62.802° W, elevation 30–200 m a.s.l.). Samples were collected under Parks Canada Research permit TMNP-2012-11533. Soils ranged from mostly loamy (samples 1 to 7) to sandy (sample 8). Boreal peat samples were collected from a poor nutrient *Sphagnum*-dominated fen at the White River Experimental Research sites near White River, Ontario, Canada (48.354° N; 85.338° W, elevation 450 m a.s.l.), managed by the Ontario Forest Research Institute and the Ontario Ministry of Natural Resources, with permission. Eight large, intact peat cores complete with above-ground herbaceous and shrubby vegetation were collected [45]. Sub-samples of peat (approximately 10 g fresh weight) were obtained from the top (0–5 cm), and bottom (~30 cm) horizon from each core. A list of associated aboveground vegetation for both sampling locations can be found in S1 Table.

### DNA extraction

Soil samples (approximately 10 g wwt) were shaken in 100 mL of 1M sodium pyrophosphate (Anachemia) to disrupt soil colloids. Samples were washed through three stacked sieves (1.18 mm, 250 um, 53 um) for 1–2 minutes with dH_2_O. Organic matter, including mycelia and spores, was collected from the finest sieve and lyophilised for 24 h (Virtis Bench Top 3.5 Freeze Dryer), then ground in liquid nitrogen using a mortar and pestle. DNA was isolated from 1 g of ground sample following the high molecular weight DNA soil protocol (http://www.borealgenomics.com/assets/Aurora-HMW-Soil-App-Note-13-01-09.pdf) using an Aurora electrophoretic nucleic acid separator (Boreal Genomics) [46]. Cartridges were washed and soaked in 10% bleach for at least 30 minutes between each sample.

Peat samples (approximately 25 g wwt) were lyophilised for 48 h, an aliquot ground in liquid nitrogen, and kept frozen until DNA extraction. Total genomic DNA was extracted from 25 mg of each sub-sample of peat moss from each horizon using a Zymo DNA isolation kit (Zymo Research Corporation) to obtain genomic DNA free of PCR-inhibitory phenolic compounds. Genomic DNA was quantified and tested for extraction quality using a nanodrop (Nanodrop 2000, Thermo Scientific) (S1 Fig). Three random high quality DNA extracts (determined by Nanodrop absorbance ratios) for both subarctic soil and upper horizon peat were chosen to be re-sequenced using ITS2 primers to allow for direct comparison of the quality and quantity of sequence data obtained by primers targeting two different regions of the rDNA gene.

### PCR protocol

Optimal PCR conditions were determined from DNA extracts of *Agaricus bisporus* and CIL Plus Mycoactive^™^ Potting Soil (for LSU200-F/LSU481-R), and of a *Harposporium* sp. isolate for LSU200A-F /LSU476A-R. LSU PCR reactions were carried out in a total volume of 25 μL, with 0.5–4 μL template DNA, 12.5 μL of Toughmix (Quanta Biosciences), and 1.25 μL each of forward and reverse primers (5 μM). Optimal PCR conditions were 2 min at 94°C, followed by 29 cycles for 30 s at 94°C, 30 s at 62°C (55°C in first cycle followed by the remainder at 62°C for Ascomycota primers), with a final elongation temperature of 72°C for 18 s. ITS2 PCR reactions followed the protocol outlined by Toju et al. [41], scaled to a total volume of 25 μL, using 1 μL template DNA (20 ng/μL for peat and soil samples). Optimal PCR conditions were 3 min at 94°C, followed by 35 cycles for 20 s at 94°C, 30 s at 47°C, 20 s at 72°C, with a final elongation temperature of 72°C for 7 min [41]. PCR products were normalized with a Qubit fluorimeter with the dsDNA HS kit (Life Technologies) and submitted for paired-end MiSeq Illumina sequencing (2 x 300 bp with V3 chemistry) at the London Regional Genomics Centre (Robarts Research Institute, London, Canada).

### Bioinformatic analysis

Two MiSeq runs were done for each LSU primer set, and for each soil type, resulting in four multiplexed runs, and separate bioinformatic analyses for each run. All ITS2 samples were run together on a separate multiplexed MiSeq run. Raw FASTQ data was sent through a custom MiSeq data processing pipeline (https://github.com/ggloor/miseq_bin/tree/master).

PANDAseq (https://github.com/neufeld/pandaseq) [47] was used to overlap reads with a minimum overlap length of 30 nt. Any sequences containing ambiguous base calls were removed, as well as any reads that did not perfectly match the primer sequence. Barcode and primer sequences were trimmed prior to clustering. The pipeline grouped the overlapped FASTQ output into identical sequences (ISUs) by identity. The ISUs were checked for chimeras using the UCHIME algorithm [48] and grouped into operational taxonomic units (OTUs) at 97% similarity, using the UCLUST clustering algorithm within USEARCH v 7.0.1090. We chose 97% similarity based on a study that established this as the optimal threshold to translate OTUs into taxonomic species when using single-linkage clustering and the V6 variable region of SSU (16S) rDNA, a region of comparable variability to the D1 divergent domain of LSU rDNA [49]. Therefore, each OTU consists of a centroid sequence—a representative sequence from the most common sequence type within each OTU—around which are clustered reads that are ≥97% similar. Sequence reads that were less than 0.1% of the total abundance in any sample were automatically removed from the sample-mapped OTU table.

Because of the large dataset obtained by NGS, more precise identification of each OTU using a phylogenetic approach and post-hoc analysis is not practical, nor required for ecological comparisons of fungal communities. Instead, general taxonomic classifications were done via the online RDP Classifier [50] using the Fungal LSU Training set 11 as a reference database (https://rdp.cme.msu.edu/classifier/classifier.jsp) for the two LSU primer sets, and the Warcup Fungal ITS Trainset 1 for the ITS2 data (https://rdp.cme.msu.edu/classifier/classifier.jsp). However, centroid sequences with confidence scores lower than 90% were queried using a nucleotide BLAST [51] and re-classified using taxa that scored >80% query coverage and identity within the distance tree of results (since top BLAST hits are not always the closest phylogenetic neighbour [52]).

Raw data can be found at the ENA website under the accession number PRJEB11433. Centroid sequences of all OTUs and occurrence of OTUs across all samples (tag-mapped OTU tables), with RDP and Blast annotations, are available as S2 Table.

## Results

### Primer performance: target sequence yields

In total, 1 435 400 reads representing 68 670 ISUs were amplified from eight subarctic soil samples using the LSU200-F/LSU481-R primers (Table 2). Of these, 4 827 were flagged as chimeric, leaving 63 843 clusters that were grouped into 2 991 OTUs at 97% similarity. Prior to analysis, 2 681 OTUs with singleton reads were discarded, leaving 310 OTUs (877 244 total reads). There were 188 fungal (target) OTUs identified (60.6% of total retained OTUs; 79.6% of final reads). From peat, many more raw reads were obtained (4 777 383); after quality filters, these clustered to 403 OTUs, of which 122 (30.3%) were identified as fungal, but the fraction of final reads on-target was similar (71.7%). Using the LSU200A-F/LSU476A-R primers, 212 target (Ascomycota) OTUs were recovered from subarctic soils (87.6% of final OTUs, 97.9% of final retained reads), and 89 Ascomycota OTUs from peat (63.5% of final OTUs, 95.3% of final retained reads). The ITS2 primers (ITS3_KYO2/ITS4_KYO3) were used on a subset of soil and peat samples, and yielded 554 358 reads, which after quality filtering, clustered to 73 OTUs, of which 72 were fungal (98.6% of final OTUs, 99.5% of final reads).

**Table 2:** Summary data for all sample sets using LSU200-F/LSU481-R, and LSU200A-F/LSU476A-R (Ascomycota) primers developed in this study, and ITS3_KYO2/ITS4_KYO3 primers developed by Toju et al. [41].

Primers	LSU200-F/LSU481-R		LSU200A-F/LSU476A-R		ITS3_KYO2/ ITS4_KYO3
**No. samples**	8 soil	16 peat	8 soil	16 peat	3 soil; 3 peat
**Total reads**	1 435 400	4 777 383	466 613	1 048 373	554 358
**ISUs**	68 670	179 317	24 862	40 705	27 116
**Chimeras (ISUs)**	4 827	8 151	1 223	3361	262
**Clustered OTUs**	2 991	2 296	772	851	1 421
**Singleton OTUs**	2 681	1 893	530	711	1 348
**Final OTUs**	310	403	242	140	73
**Final reads**	877 244	3 741 924	314 962	802 078	235 801
**Target (fungal) OTUs**	188	122	212	89	72
**Percent target OTUs**	60.60%	30.30%	87.60%	63.50%	98.60%
**Target reads as % of final reads**	79.60%	71.70%	97.90%	95.30%	99.45%
**Mean target reads per sample**	87 286	167 685	38 543	47 774	39 084
**Mean OT Score**^a^**, %**	48.6	56.2	66.1	72.9	42.3
**Percent non-target OTUs**	39.40%	69.70%	12.40%	36.40%	1.40%
**Percent non-target reads**	20.40%	28.3	2.10%	4.60%	0.55%

^a^ The percentage of raw reads per sample retained after quality filtering and on-target

The number of raw reads and of on-target reads per sample is dependent in part on the yield of the Illumina run and the number of samples multiplexed per run, but the latter number is useful as a judge of sequencing depth for each primer-sample pairing. The mean number of on-target reads per sample ranged from 38 543 to 167 685; values for the reactions using ITS2 primers were intermediate (Table 2). An informative measure of primer performance is the OT Score, the percent of raw reads per sample retained after quality filtering, and on target. The mean OT Scores for the three primer sets in our tests ranged from 42.3 for the ITS2 primers to 72.9 for the LSU200A-F/LSU476A-R primers with peat samples.

### Primer performance: fungal diversity recovered

The diversity of target and non-target OTUs recovered using the new the LSU200-F/LSU481-R and LSU200A-F/LSU476A-R primers from the full set of eight soil and 16 peat samples is available through the European Nucleotide Archive (ENA) under the accession number PRJEB11433. Here, we present the diversity recovered from the subset of samples that were sequenced using both these primers and the ITS2 primers. Using ITS2 primers, the 72 fungal OTUs included 19 of Basidiomycota and 53 of Ascomycota; 34 of these 72 OTUs could be placed with confidence in 29 genera, and 19 OTUs could be identified to species (Table 3). Using the LSU200-F/LSU481-R primers, 158 OTUs could be placed in 79 genera and 27 identified to species, and using LSU200A-F/LSU476A-R primers, 90 OTUs could be identified to 64 genera, and 21 identified to species. Together, the LSU primers recovered 127 genera and 28 species that were not obtained using the ITS2 primers, but the ITS2 primers recovered 10 unique genera and 16 species that were not obtained using either of the LSU primers.

**Table 3:** List of genera and species recovered by LSU200-F/LSU481-R, LSU200A-F/LSU476A-R and ITS3_KYO2/ITS4_KYO3 primers. Taxa were included only if the total read count across the three subsamples were >3, and are listed alphabetically by higher taxa (A = Ascomycota, B = Basidiomycota, C = Chytridiomycota, E = Entomophthoromycota, G = Glomeromycota, K = Kickxellomycotina, M = Mucoromycotina, Mi = Microsporidia, Z = Zoopagomycotina). (#) = Number of OTUs identified to that taxon.

LSU200-F/ LSU481-R	LSU200A-F/ LSU476A-R	ITS3_KYO2/ ITS4_KYO3
B *Agaricus* sp. (2)	A *Acephala applanata*	A *Acephala* sp.
B *Amanita* sp.	A *Alternaria* sp.	A *Cenococcum geophilum* (2)
B *Amphinema* sp.	A *Ascocoryne turficola*	A *Cladonia* sp.
B *Arcangeliella* sp.	A *Babjevia anomalus*	A *Cladophialophora sp. (2)
B *Asterostroma laxum*	A *Byssonectria* sp.	A *Colpoma sp.
B *Athelia epiphylla*	A *Capronia* sp.	A *Hyaloscypha albohyalina
B *Athelia* sp.	A *Caproventuria hanliniana* (2)	A *Hymenoscyphus* sp. (2)
B *Basidiodendron* sp. (3)	A *Cenococcum* sp. (3)	A *Leptodontidium elatius
B *Brevicellicium exile*	A *Cladonia* sp.	A *Meliniomyces variabilis
B *Caloboletus inedulis*	A *Cladosporium*	A *Phialocephala fortinii
B *Ceratobasidium* sp.	A *Clonostachys rosea*	A *Remleria rhododendricola
B *Clavaria* aff. *fragilis*	A *Collophora* sp.	A *Rhizoscyphus ericae
B *Clavaria* sp. (5)	A *Coniochaeta* sp.	A *Stereocaulon tomentosum*
B *Clavulina* sp.	A *Cryptodiscus microstomus*	
B *Conocybe* sp.	A *Cryptodiscus rhopaloides*	B *Ceratobasidium* sp.
B *Coprinellus* sp.	A *Dactylaria* sp. (2)	B *Clavaria* sp. (2)
B *Coprinopsis* sp. (2)	A *Davidiella* sp. (2)	B *Inocybe acutoides*
B *Cortinarius* aff. *fragilis*	A *Devriesia* sp.	B *Leccinum rotundifoliae*
B *Cortinarius* sp. (5)	A *Diplococcium spicatum* (2)	B *Mycena alexandri*
B *Craterellus* sp.	A *Dipodascopsis* sp.	B *Mycena galericulata*
B *Cryptococcus gilvescens*	A *Doratomyces* sp.	B *Mycena* sp.
B *Cryptococcus magnus*	A *Elaphocordyceps* sp. (2)	B *Pseudotomentella tristis
B *Cryptococcus musci*	A *Elaphomyces* sp.	B *Ramariopsis* sp.
B *Cryptococcus* sp.	A *Exophiala* sp.	B *Russula aeruginea*
B *Cryptococcus terricola* (2)	A *Fusicladium* sp.	B *Sebacina* sp. (2)
B *Cuphophyllus* sp.	A *Gaeumannomyces* sp.	B *Serendipita vermifera*
B *Cymatoderma* sp.	A *Geoglossum sphagnophilum*	B *Sistotrema oblongisporum
B *Entoloma* sp.	A *Glonium* sp.	B *Suillus cavipes*
B *Entoloma strictius*	A *Godronia* sp.	B *Tomentella* sp.
B *Exidia saccharina* (2)	A *Heleiosa* sp.	B *Tylospora fibrillosa*
B *Exidia* sp. (2)	A *Hemileucoglossum alveolatum* (3)	
B *Exobasidium* sp.	A *Hymenoscyphus* sp.	
B *Galerina paludosa*	A *Lachnum* sp.	
B *Galerina* sp. (2)	A *Lecanicillium* sp. (2)	
B *Guehomyces* sp.	A *Leucophagus* sp. (2)	
B *Hygrocybe* sp. (5)	A *Maasoglossum* sp.	
B *Hyphoderma* sp. (2)	A *Melastiza* sp. (3)	
B *Hyphodontia* sp. (2)	A *Metarhizium* sp.	
B *Inocybe borealis*	A *Mitrula* sp. (2)	
B *Inocybe catalaunica*	A *Mniaecia jungermanniae* (2)	
B *Inocybe* sp. (3)	A *Mniaecia gloeocapsae*	
B *Inocybe splendens*	A *Mollisia* sp. (3)	
B *Inocybe squarrosa*	A *Morchella* sp.	
B *Jaapia* sp.	A *Myxotrichum* sp.	
B *Laccaria* sp.	A *Neofabraea* sp.	
B *Lactarius* sp.	A *Oidiodendron* sp. (4)	
B *Leccinum* sp.	A *Penicillium* sp. (5)	
B *Leucosporidium* sp.	A *Peziza* sp.	
B *Limonomyces* sp.	A *Phialophora* sp.	
B *Lycoperdon* sp.	A *Pochonia* sp.	
B *Mrakia* sp.	A *Preussia* sp.	
B *Mycena* sp. (3)	A *Pseudoplectania episphagnum*	
B *Mycogloea* sp.	A *Repetophragma* sp.	
B *Nidula* sp.	A *Rhizosphaera* sp.	
B *Phanerochaete* sp.	A *Sabuloglossum arenarium*	
B *Postia* sp.	A *Sorocybe* sp. (2)	
B *Psathyrella* sp.	A *Sphaeropezia* sp.	
B *Ramariopsis* sp. (5)	A *Spirosphaera minuta*	
B *Russula* sp.	A *Stereocaulon* sp.	
B *Sebacina* sp. (9)	A *Stomiopeltis* sp.	
B *Serendipita vermifera*	A *Strelitziana cliviae*	
B *Spiculogloea* sp.	A *Thelebolus* sp.	
B *Sporobolomyces* sp.	A *Trichoderma* sp.	
B *Suillus cavipes*	A *Troposporella* sp.	
B *Suillus palustris*	A *Umbilicaria* sp.	
B *Syzygospora* sp.		
B *Tephrocybe* sp.		
B *Tilletia* sp.		
B *Tomentella* sp. (3)		
B *Tomentellopsis* sp.		
B *Tremella* sp.		
B *Tricholoma* sp.		
B *Tulasnella* sp. (2)		
B *Tylospora* sp. (3)		
B *Udeniomyces* sp.		
B *Xerocomus* sp.		
B *Xylodon nespori*		
C *Betamyces* sp.		
C *Chytridium* sp. (3)		
C *Fayochytriomyces spinosus*		
C *Gonapodya* sp.		
C *Irineochytrium annulatum* (2)		
C *Monoblepharis* sp.		
C *Olpidium* sp.		
E *Basidiobolus* sp.		
G *Archaeospora* sp. (2)		
G *Glomus* sp. (3)		
K *Spiromyces* sp. (2)		
K *Stachylina* sp.		
M *Endogone* sp. (4)		
M *Mortierella globulifera* (5)		
M *Mortierella* sp. (12)		
M *Umbelopsis* sp. (6)		
Mi *Mitosporidium daphniae*		
Z *Rhopalomyces* sp.		

* Unique genera identified by ITS2 primers (ITS3_KYO2/ITS4_KYO3)

There was broad agreement at higher taxonomic levels between the fungal communities detected using the LSU primers and ITS2 primers (S3 Table). The predominant fungi recovered from subarctic soils using LSU200-F/LSU481-R primers included Mortierellomycetes, Agaricales, Thelephorales, and Sebacinales; OTUs recovered using LSU200A-F/LSU476A-R primers were dominated by members of Helotiales, Leotiomycetes, and Chaetothyriales; OTUs recovered using ITS2 primers by Helotiales, unknown Pezizomycotina, Chaetothyriales, Agaricales, Russulales, Sebacinales and Thelephorales. From peat, the OTUs recovered using LSU200-F/LSU481-R primers were dominated by Mortierellomycetes, Agaricales, Atheliales, Cantharellales, and Sebacinales; those recovered using LSU200A-F/LSU476A-R primers by Helotiales, unknown Ascomycota, and Mytilinidiales; and those recovered using ITS2 primers by Helotiales, unknown Leotiomycetes, Agaricales, Chaetothyriales and Sebacinales.

## Discussion

The primer sets presented here were designed to provide high efficiency sequence tag information data on fungal diversity in Next Generation Sequencing studies using the Illumina platform. Both primer sets (LSU200-F/LSU481-R and LSU200A-F/LSU476A-R) amplify a ~300 bp region of the rDNA LSU, which provides reliable placements of sequence clusters within the Fungi and, together they yield a broad array of Fungi from chytrids to mushrooms. In addition, the non-target reads obtained using the LSU200-F/LSU481-R primers (approximately 20–30% of retained reads) provide sequence data on a number of important eukaryotic groups that are poorly known in soil [53]: Amoebozoa, Centroheliozoa, Choanoflagellida, Metazoa, Rhizaria and unicellular Streptophyta. We establish the validity and reproducibility of these primers for high-throughput sequencing of environmental samples using replicates of DNA extracted from two types, one of which (peat) was particularly high in humic acid and phenolic compounds.

Including both soil and peat samples, data clean-up resulted in the loss of 25.7% of reads, on average, for primers LSU200-F/LSU481-R, and 26.3%, on average, using the LSU200A-F/LSU476A-R primers. The complement of the data lost to poor quality sequences, chimeras, singleton OTUs, and non-target sequences is the OT Score, the percent of raw sequence reads retained and on-target. The OT Scores for LSU200-F/LSU481-R primers averaged 53.7 across the samples in our study, and 70.6 for LSU200A-F/LSU476A-R primers. These values are higher than the OT Score for the ITS2 primers ITS3_KYO2/ITS4_KYO3 of 42.3 (57.5% data loss to clean-up) in this study, and considerably better than the 78.6% data loss reported for those primers in [54]. In our study, the greatest loss of reads occurred through elimination of singleton OTUs, discarded from our data prior to analysis. Singleton reads are often removed to avoid introducing biases in OTU computation, since poor sequences—reads with greater numbers of errors—are likely to inflate the number of OTUs recovered [55]. In contrast, the inclusion of singletons is unlikely to change observed data trends in ecological comparisons even if they include true observations of rare species [56]. While our data had high loss of OTUs after the removal of singletons, read loss—which is arguably a more important limitation in metagenomic studies [57]—was minimal.

Although the ITS2 primers recovered as many on-target sequences per sample as our new LSU primers in our tests, they yielded far fewer fungal OTUs, representing a much smaller range of higher taxa of fungi than were obtained using our new primers LSU200-F/LSU481-R and LSU200A-F/LSU476A-R. The only other published study using these ITS2 primers for the purpose of biodiversity discovery on DNA extracted from soil samples [58] recovered considerably more total fungal OTUs (3208 OTUs) than our data (72 OTUs). However, Balint et al. [58] analysed considerably more samples (96 versus 6), used two different annealing temperatures (51 and 55°C), and ran three PCR replications for each annealing temperature. This suggests that three replicates of two different annealing temperatures and a larger number of target reads may be required for the ITS2 primers to obtain a similar number OTUs as our new LSU primers [54]. That said, the ITS2 primers, which were designed to recover Ascomycota and Basidiomycota [41], will not yield sequences of chytrids, Glomeromycota, or the zygomycete groups obtained using LSU200-F/LSU481-R, and in our tests they also failed recover important groups of Ascomycota (e.g., Eurotiales, Mytilinidiales, Pezizales, Saccharomycetales, Venturiales) and Basidiomycota (e.g., Exobasidiomycetes, Microbotryomycetes, Pucciniomycetes, and Tremellomycetes) that were recovered by our primers. In contrast, the ITS2 primers did recover two OTUs tentatively identified as Archaeorhizomycetes (Ascomycota), an important group in boreal/montane soils and peat [59] not recovered using LSU200A-F/LSU476A-R. However, as noted in Table 1, improving the match of primer LSU200A-F to Archaeorhizomycetes may be achieved by using the modified forward primer AACKGCGAGTGAAGCRGBM; the reverse primer LSU476A-R already has a perfect match to known Archaeorhizomycetes sequences.

Primer specificity, or the lack of primer universality in recovering broad groups such as the entire kingdom Fungi, has been a persistent issue that has not previously been addressed for biodiversity studies using the Illumina platform. With the exception of LR3/TW13 and LR5/TW14 primers that have been designed based on a highly conserved region within the LSU, all other fungal specific genetic markers and ‘universal’ primers discriminate against various groups of fungi [60]. The ITS1F and other ITS primers (ITS1 and ITS5) have shown biases towards the amplification of fungal groups within the Basidiomycota, while others, particularly ITS2, ITS3 and ITS4, favour Ascomycota [60]. More specific primers such as the ITS4-B, designed to capture basidiomycetes, can amplify the ITS region of a very limited group within the target phylum [61]. Since our new LSU primers provide ample read counts in environmental samples, have low rates of data loss, and cover a breadth of fungal taxa, we suggest that they provide a promising addition to complement and counterpart other universal primers in metagenomic studies.

## Supporting Information

S1 Fig DNA concentrations and comparative absorption values for three soil types. Black lines indicate the median, red diamonds indicate the mean. (A) DNA concentrations (ng/μL) of three soil types after Aurora HMW DNA Extraction (Subarctic Soil), and Zymogen Soil DNA Extraction Kit (Lower Peat and Upper Peat). (B) 260/280 and (C) 260/230 absorption ratios for the resulting DNA extractions. A ratio value of ~1.8 for 260/280 and 2.0–2.2 for 260/230 values indicate DNA free from most proteins, phenols, or other contaminants.
(DOCX)

S1 Table Partial list of vegetation found in association with samples taken from subarctic and peatland soils. Peat plant species list obtained from [Dieleman et al. [44]] Supplementary Table S1; plants associated with subarctic soil samples identified by Michael Burzynski.
(DOCX)

S2 Table Retrieved sequence data from all samples (OTU tag-mapped tables), using primer sets LSU200A-F/LSU476A-R (Ascomycota), LSU200-F/LSU481-R (all other fungi), and ITS3_KYO2/ITS4_KYO3, including RDP and BLASTN annotations. (XLSX)

S3 Table Median relative frequency and range (median; range) of sequences amplified by new LSU primers in comparison to Toju et al. (2012) ITS2 primers. Relative frequency (%) calculated using total number of reads for each soil type (348 237^a^, 941 098^b^, 134 391, and 96 830 reads, respectively; n = 3 for all).
(DOCX)

## Abbreviations

BLAST: Basic Local Alignment Search Tool
D1: variable Domain 1
ITS: Internal Transcribed Spacer
ISU: Identical Sequence Units
LSU: Large Subunit
NCBI: National Center for Biotechnology Information
NGS: Next Generation Sequencing
OTU: Operational Taxonomic Unit
PCR: Polymerase Chain Reaction
rDNA: ribosomal DNA
