# Supplementary material for: New Primers for Discovering Fungal Diversity Using Nuclear Large Ribosomal DNA
Source: PLoS One. 2016 Jul 8;11(7):e0159043. doi: 10.1371/journal.pone.0159043 (PMC4938210; doi:10.1371/journal.pone.0159043)

**S1 Figure. DNA concentrations and comparative absorption values for three soil types.**

Black lines indicate the median, red diamonds indicate the mean. (A) DNA concentrations (ng/µL) of three soil types after Aurora HMW DNA Extraction (Subarctic Soil), and Zymogen Soil DNA Extraction Kit (Lower Peat and Upper Peat). (B) 260/280 and (C) 260/230 absorption ratios for the resulting DNA extractions. A ratio value of ~1.8 for 260/280 and 2.0–2.2 for 260/230 values indicate DNA free from most proteins, phenols, or other contaminants.


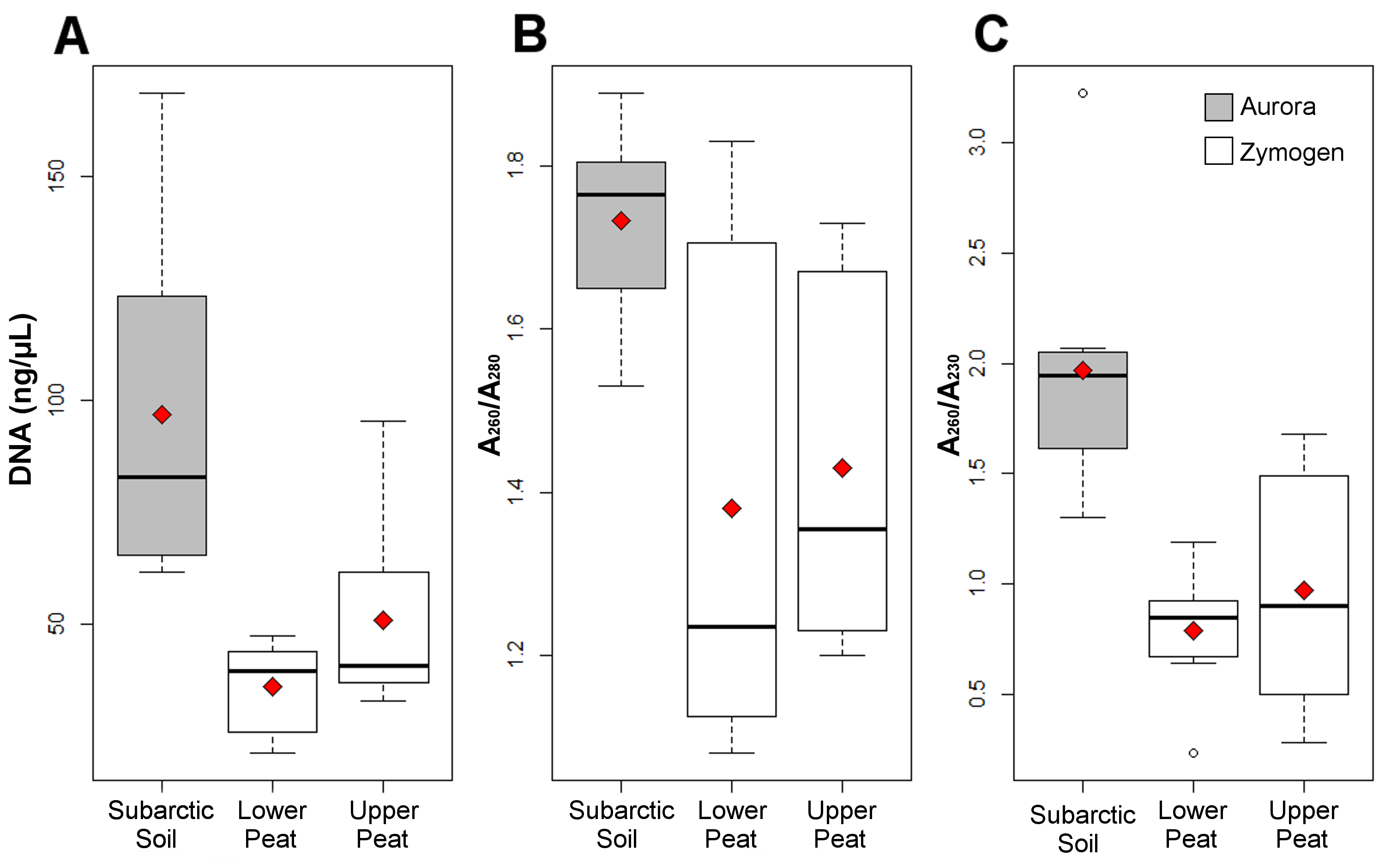

Supplement: S1 Fig — Black lines indicate the median, red diamonds indicate the mean. (A) DNA concentrations (ng/μL) of three soil types after Aurora HMW DNA Extraction (Subarctic Soil), and Zymogen Soil DNA Extraction Kit (Lower Peat and Upper Peat). (B) 260/280 and (C) 260/230 absorption ratios for the resulting DNA extractions. A ratio value of ~1.8 for 260/280 and 2.0–2.2 for 260/230 values indicate DNA free from most proteins, phenols, or other contaminants. (DOCX) [file pone.0159043.s001.docx]
