# Supplementary material for: New Primers for Discovering Fungal Diversity Using Nuclear Large Ribosomal DNA
Source: PLoS One. 2016 Jul 8;11(7):e0159043. doi: 10.1371/journal.pone.0159043 (PMC4938210; doi:10.1371/journal.pone.0159043)
Supplement: S1 Table — Peat plant species list obtained from [Dieleman et al. [44]] Supplementary Table S1; plants associated with subarctic soil samples identified by Michael Burzynski. (DOCX) [file pone.0159043.s002.docx]

**S1 Table. Partial list of vegetation found in association with samples taken from subarctic and peatland soils.** Peat plant species list obtained from [[Dieleman et al. [44]](#_ENREF_44)] Supplementary Table S1; plants associated with subarctic soil samples identified by Michael Burzynski.

| **Subarctic soils in Torngat, Labrador** | **^1^Peatland soils in White River, ON** |
| --- | --- |
| *Armeria maritima* | *Abies balsamea* |
| *Antennaria* sp. | *Andromeda polifolia* |
| *Arnica angustifolia* subsp. *angustifolia* | *Campylium stellatum* var. *stellatum* |
| *Astragalus alpinus* | *Carex disperma* |
| *Betula glanduosa* | *Carex magellanica* |
| *Calamagrostis stricta* | *Chamaedaphne calycalata* |
| *Carex scirpoidea* | *Drosera rotundifolia* |
| *Cerastium arvense* | *Gaultheria hispidula* |
| *Empetrum nigrum* | *Kalmia polifolia* |
| *Eriophorum* sp. |  |
| *Pedicularis labradorica* | *Lycopodiella inundata* |
| *Poa* sp. | *Maianthemum trifolium* |
| *Potentilla crantzii* | *Picea mariana* |
| *Phyllodoce caerulea* | *Rhododendron groenlandicum* |
| *Rhodiola rosea* | *Sphagnum* spp. |
| *Rhododendron groenlandicum* | *Vaccinium myrtilloides* |
| *Rhododendron tomentosum* subsp. *subarcticum* | *Vaccinium oxycoccos* |
| *Salix* sp. |  |
| *Solidago multiradiata* |  |
| *Taraxacum* sp. |  |
| *Vaccinium uliginosum* |  |

^1^ Plant species list obtained from ([Dieleman et al., 2015](#_ENREF_8)) Supplementary Table S1.
