# Supplementary material for: New Primers for Discovering Fungal Diversity Using Nuclear Large Ribosomal DNA
Source: PLoS One. 2016 Jul 8;11(7):e0159043. doi: 10.1371/journal.pone.0159043 (PMC4938210; doi:10.1371/journal.pone.0159043)
Supplement: S3 Table — Relative frequency (%) calculated using total number of reads for each soil type (348 237a, 941 098b, 134 391, and 96 830 reads, respectively; n = 3 for all). (DOCX) [file pone.0159043.s004.docx]

**S3 Table. Median relative frequency and range (median; range) of sequences amplified by new LSU primers in comparison to Toju et al. (2012) ITS2 Fungal primers.** Relative frequency (%) calculated using total number of reads within the phylum for each soil type (348 237^a^, 941 098^b^, 134 391, and 96 830 reads, respectively; n=3 for all).

| **Rank^c^** | **LSU primers** | | | | **ITS2 Fungal primers** | | | |
| --- | --- | --- | --- | --- | --- | --- | --- | --- |
|  | **Subarctic soil** | | **Upper peat** | | **Subarctic soil** | | **Upper peat** | |
| Fungi: Unknown | 0.22 | 0.33 | | 0.23 | | 1.66 | |  |
| Zygomycota: Kickxellomycotina:  Kickxellales | 0.00 | — | | — | | — | |  |
| Z: K: Harpellales | — | 0.00 | | — | | — | |  |
| Z: Mucoromycotina: Unknown | 0.30 | — | | — | | — | |  |
| Z: Muc: Mucorales | 0.48 | 0.17 | | — | | — | |  |
| Z: Muc: Endogonales | 0.26 | 0.00 | | — | | — | |  |
| Z: Muc: Mortierellales | 11.30 | 3.88 | | — | | — | |  |
| Z: Entomophthoromycotina | — | 0.04 | | — | | — | |  |
| Z: Zoopagomycotina | — | 0.71 | | — | | — | |  |
| Chytridiomycota: Unknown | — | 1.07 | | — | | — | |  |
| C: Chytridiomycetes: Unknown | 0.39 | 1.54 | | — | | — | |  |
| C: Chytr: Chytridiales | 0.32 | 0.08 | | — | | — | |  |
| C: Chytr: Rhizophydiales | 0.14 | — | | — | | — | |  |
| C: Chytr: Spizellomycetales | 0.00 | 0.03 | | — | | — | |  |
| C: Chytr: Lobulomycetales | 0.15 | 0.02 | | — | | — | |  |
| C: Monoblepharidomycetes:  Monoblepharidales | 0.05 | 0.04 | | — | | — | |  |
| Glomeromycota: Unknown | — | 0.06 | | — | | — | |  |
| G: Glomeromycetes: Archaeosporales | 4.46 | — | | — | | — | |  |
| G: Glom: Glomerales | 1.62 | — | | — | | — | |  |
| Basidiomycota: Unknown | 0.73 | 0.30 | | 0.95 | | 0.00 | |  |
| B: Agaricostilbomycetes: Spiculogloeales | — | 0.05 | | — | | — | |  |
| B: Pucciniomycetes: Unknown | 0.08 | — | | — | | — | |  |
| B: Microbotryomycetes incertae sedis | 0.05 | 0.05 | | — | | — | |  |
| B: M: Leucosporidiales | 0.06 | — | | — | | — | |  |
| B: M: Sporidiobolales | 0.00 | — | | — | | — | |  |
| B: Exobasidiomycetes: Exobasidiales | 0.05 | — | | — | | — | |  |
| B: Exobasidiomycetes: Tilletiales | — | 0.00 | | — | | — | |  |
| B: Tremellomycetes: Unknown | 0.07 | — | | — | | — | |  |
| B: T: Cystofilobasidiales | 0.15 | — | | — | | — | |  |
| B: T: Filobasidiales | 1.09 | 0.00 | | — | | — | |  |
| B: T: Tremellales | 0.07 | 0.02 | | — | | — | |  |
| B: Agaricomycetes: Unknown | 3.01 | 1.40 | | — | | — | |  |
| B: Ag: Auriculariales | 0.80 | 0.12 | | — | | — | |  |
| B: Ag: Sebacinales | 2.82 | 8.30 | | 3.81 | | 2.82 | |  |
| B: Ag: Cantharellales | 2.28 | 5.81 | | 0.01 | | 1.08 | |  |
| B: Ag: Trechisporales | 0.28 | — | | — | | — | |  |
| B: Ag: Gomphales | — | 0.00 | | — | | — | |  |
| B: Ag: Hymenochaetales | 0.02 | 3.12 | | — | | — | |  |
| B: Ag: Corticiales | — | 2.24 | | — | | — | |  |
| B: Ag: Polyporales | 0.04 | 3.41 | | 0.00 | | 0.61 | |  |
| B: Ag: Thelephorales | 7.11 | 0.48 | | 5.34 | | 0.02 | |  |
| B: Ag: Russulales | 1.03 | 0.00 | | 13.37 | | 0.03 | |  |
| B: Ag: Agaricales | 8.98 | 30.15 | | 11.90 | | 6.57 | |  |
| B: Ag: Atheliales | 0.04 | 12.68 | | — | | — | |  |
| B: Ag: Boletales | 1.88 | 2.18 | | 0.87 | | 0.41 | |  |
| Ascomycota: Unknown | 2.51 | 2.24 | | — | | — | |  |
| Ascomycota incertae sedis | 0.13 | 0.04 | | — | | — | |  |
| A: Saccharomycetes:  Saccharomycetales | 0.33 | 0.04 | | — | | — | |  |
| A: Orbiliomycetes: Orbiliales | 0.01 | 0.02 | | — | | — | |  |
| A: Archaeorhizomycetes^d^ | — | — | | 0.53 | | 0.55 | |  |
| A: Pezizomycotina: Unknown | — | — | | 5.13 | | 0.67 | |  |
| A: Pezizomycetes: Pezizales | 0.07 | 0.08 | | — | | — | |  |
| A: Geoglossomycetes: Geoglossales | 0.02 | 0.10 | | — | | — | |  |
| Ascomycota: Coniocybomycetes:  Coniocybales | 0.05 | — | | — | | — | |  |
| Ascomycota: Dothideomycetes:  Unknown | 0.05 | — | | — | | — | |  |
| A: D: incertae sedis | 2.30 | — | | 2.90 | | 0.06 | |  |
| A: D: Capnodiales | 0.41 | 0.00 | | — | | — | |  |
| A: D: Pleosporales | 0.39 | 0.05 | | — | | — | |  |
| A: D: Mytilinidiales | 0.44 | 1.50 | | — | | — | |  |
| A: D: Venturiales | 3.25 | 0.04 | | — | | — | |  |
| A: Eurotiomycetes: Unknown | 0.25 | — | | 2.67 | | 0.03 | |  |
| A: E: Chaetothyriales | 3.40 | 0.19 | | 4.13 | | 2.92 | |  |
| A: E: Eurotiales | 1.16 | 0.20 | | — | | — | |  |
| A: Lecanoromycetes: Unknown | 1.00 | — | | — | | — | |  |
| A: Lecanoromycetes: Lecanorales | 3.17 | — | | 3.35 | | 0.04 | |  |
| A: Lecanoromycetes: Ostropalales | 0.02 | — | | — | | — | |  |
| A: Leotiomycetes: Unknown | 6.29 | 0.28 | | 0.23 | | 5.88 | |  |
| A: Leotiomycetes: incertae sedis | 0.63 | 0.13 | | 0.12 | | 3.37 | |  |
| A: L: Helotiales | 20.15 | 16.41 | | 42.28 | | 71.12 | |  |
| A: L: Rhytismatales | 1.17 | 0.01 | | 2.14 | | 0.44 | |  |
| A: L: Thelebolales | 0.45 | — | | — | | — | |  |
| A: Sordariomycetes: Unknown | 1.01 | 0.00 | | — | | — | |  |
| A: S: incertae sedis | 0.30 | — | | — | | — | |  |
| A: S: Xylariales | 0.01 | — | | 0.01 | | 0.54 | |  |
| A: S: Hypocreales | 0.66 | 0.37 | | 0.02 | | 1.18 | |  |
| A: S: Microascales | 0.00 | 0.00 | | — | | — | |  |
| A: S: Coinochaetales | 0.04 | — | | — | | — | |  |
| A: S: Diaporthales | 0.01 | — | | — | | — | |  |
| A.S: Sordariales | — | 0.02 | | — | | — | |  |
| A: S: Magnaporthales | 0.00 | 0.00 | | — | | — | |  |

^a^ Summed read total of both general fungal (167 174 reads) and Ascomycota-specific primers (181 063 reads) for three subarctic soil samples

^b^ Summed read total of both general fungal (746 453 reads) and Ascomycota-specific primers (194 645 reads) for three upper peat samples

^c^ Acronyms for phyla and classes: A – Ascomycota, Ag – Agaricomycetes, B – Basidiomycota, C – Chytridiomycota, Chytr – Chytridiomycetes, D – Dothideomycetes, E – Eurotiales, Glom – Glomeromycetes, K – Kickxellomycotina, Lec – Lecanoromycetes, Leo – Leotiomycetes, M – Microbotryomycetes, Muc – Mucoromycotina, S – Sordariomycetes, T – Tremellomycetes, Z – Zygomycota.

^d^ Archaeorhizomycetes or allies (Ascomycota)
